# Comparison of Three Targeted Enrichment Strategies on the SOLiD Sequencing Platform

**DOI:** 10.1371/journal.pone.0018595

**Published:** 2011-04-29

**Authors:** Dale J. Hedges, Toumy Guettouche, Shan Yang, Guney Bademci, Ashley Diaz, Ashley Andersen, William F. Hulme, Sara Linker, Arpit Mehta, Yvonne J. K. Edwards, Gary W. Beecham, Eden R. Martin, Margaret A. Pericak-Vance, Stephan Zuchner, Jeffery M. Vance, John R. Gilbert

**Affiliations:** 1 John T. MacDonald Department of Human Genetics, Hussman Institute for Human Genomics, University of Miami Miller School of Medicine, Miami, Florida, United States of America; 2 Life Technologies, Inc., Carlsbad, California, United States of America; Tel Aviv University, Israel

## Abstract

Despite the ever-increasing throughput and steadily decreasing cost of next generation sequencing (NGS), whole genome sequencing of humans is still not a viable option for the majority of genetics laboratories. This is particularly true in the case of complex disease studies, where large sample sets are often required to achieve adequate statistical power. To fully leverage the potential of NGS technology on large sample sets, several methods have been developed to selectively enrich for regions of interest. Enrichment reduces both monetary and computational costs compared to whole genome sequencing, while allowing researchers to take advantage of NGS throughput. Several targeted enrichment approaches are currently available, including molecular inversion probe ligation sequencing (MIPS), oligonucleotide hybridization based approaches, and PCR-based strategies. To assess how these methods performed when used in conjunction with the ABI SOLID3+, we investigated three enrichment techniques: Nimblegen oligonucleotide hybridization array-based capture; Agilent SureSelect oligonucleotide hybridization solution-based capture; and Raindance Technologies' multiplexed PCR-based approach. Target regions were selected from exons and evolutionarily conserved areas throughout the human genome. Probe and primer pair design was carried out for all three methods using their respective informatics pipelines. In all, approximately 0.8 Mb of target space was identical for all 3 methods. SOLiD sequencing results were analyzed for several metrics, including consistency of coverage depth across samples, on-target versus off-target efficiency, allelic bias, and genotype concordance with array-based genotyping data. Agilent SureSelect exhibited superior on-target efficiency and correlation of read depths across samples. Nimblegen performance was similar at read depths at 20× and below. Both Raindance and Nimblegen SeqCap exhibited tighter distributions of read depth around the mean, but both suffered from lower on-target efficiency in our experiments. Raindance demonstrated the highest versatility in assay design.

## Introduction

While the introduction of 2^nd^ Generation sequencing has brought about a precipitous decline in per-nucleotide cost of sequencing, whole genome sequencing currently remains prohibitively expensive for the majority of study designs. Association-based studies of common genetic disorders require hundreds, if not thousands, of samples to achieve adequate statistical power. In order to take advantage of the benefits of 2^nd^ generation sequencing throughput in a cost-effective manner, many researchers are now opting to restrict the input to sequencing platforms to a subset of the full genome. In combination with the indexing and pooling of samples, targeted genomic enrichment allows for the sequencing of a smaller fraction of the genome across a much larger numbers of individuals (reviewed in [1]). Traditional methods of enriching for (or “capturing”) specific genomic regions, such as standard PCR, lack the necessary throughput to provide an efficient front-end input strategy for 2^nd^ generation sequencing platforms. To address the need for higher-throughput means of genomic selection, several targeted enrichment methods have been developed. These methods can be generally categorized into those that rely on either capture of genomic regions of interest through hybridization with oligonucleotide libraries [2]–[5], and those that use highly multiplexed PCR-based approaches (e.g. [6]). In some instances, long range PCR (LR-PCR) can also be an effective, low-cost means of providing input to 2^nd^ generation sequencers [7]–[9], but continued gains in sequencing platform throughput make this approach an increasingly inefficient front end solution. The performance metrics of each of these enrichment strategies has previously been investigated, but there is currently limited data on the use of these platforms in conjunction with the Applied Biosystems SOLiD platform. There are also very limited data resulting from multiple enrichment strategies targeting identical genomic regions, although a recent comparison was made available for the Illumina platform [10]. Here we examine three capture methods, Agilent SureSelect solution hybridization, Nimblegen SeqCap array-based hybridization, and massively parallel PCR via Raindance Technology for use in conjunction with SOLiD sequencing. A common set of genomic regions, totalling ∼0.8 Mb, was targeted by all three enrichment approaches. We examine the relative performance across a range of metrics, including targeting efficiency, replicability of performance across heterogeneous DNA samples, uniformity of coverage, and genotype concordance with independently derived genotype data from Illumina Infinium 1M arrays.

## Methods

### Sample Sources

#### Ethics statement

Written informed consent for genetic studies was obtained prior to initiating this study in agreement with protocols approved by the institutional review board (IRB) at the University of Miami Miller School of Medicine (protocol # 20070380).

#### Sample selection

In total, 18 unique human samples (11 females and 7 males) were used in this study. Blood from 16 individuals was previously collected as part of an institutional review board (IRB) approved research study (3P50NS071674-01S1), 11 of which were selected because they had genotyping data available from the Illumina 1M Infinium array. DNA was extracted from peripheral blood leukocytes using the Autopure (Gentra) automated nucleic acid extraction robotic system. Samples were further treated with RNAse-A and Proteinase K to remove remaining RNA and proteins. Two additional DNA samples were derived from anonymized human cell line DNA (Coriell); two were extracted via the Autopure automated system, and the remaining four were extracted with the Quiagen QIAamp DNA Mini Kit (Catalogue #51304). Sample sources and enrichment treatmens are outlined in Table 1 below.

**Table 1:** Enrichment methods performed for each sample.

sample_id	source	Agilent SureSelect	Nimbelgen SeqCap	Raindance
*Paired Sample Set*				
s1	blood	Y	Y	Y
s2	blood	Y	Y	Y
s3	blood	Y	Y	Y
s4	blood	Y	Y	Y
s5	blood	Y	Y	Y
s6	blood	Y	Y	Y
*Unpaired Sample Set*				
s7	blood	Y	N	N
s8	blood	N	Y	N
s9	blood	N	N	Y
s10	blood	N	N	Y
s11	blood	N	N	Y
s12	blood	N	N	Y
s13	blood	Y	Y	N
s14	blood	Y	Y	N
s15	cell	Y	Y	N
s16	cell	Y	Y	N
s17	blood	Y	N	Y
s18	blood	N	Y	Y

### Target Selection

#### Selection of target regions

Regions for targeted resequencing were selected based on two independent methods. The majority of genic regions (n = 509) were randomly sampled from the UCSC Known Genes annotation (hg18) to provide a diverse, representative set of genic targets. A smaller subset of 24 genes were specifically chosen for sequencing due to their relevance to ongoing research projects. The complete list of targeted genes is provided in Tables S1, S2, and S3. Final designs for each enrichment platform are provided in Tables S4,S5, and S6. At each selected gene locus, several genetic features were targeted for resequencing. These included 5 kb upstream of the transcription start site, all known exons, and additional evolutionarily conserved sequences. Evolutionary conservation status was established by Phasta 17-way Conserved element annotation (hg18). Target selection was conducted so as to mirror a scenario where numerous interspersed regions of interest from a genome-wide association study (GWAS) have been identified for resequencing. A common set of genomic segments (totalling ∼0.8 Mb) was targeted by all three enrichment strategies. Genomic positions (hg18 coordinates) for basepairs targeted by all three enrichment platforms are provided in Table S7. As the commercially available enrichment options from each vendor (Roche-Nimblegen, Raindance, Agilent) at the time of the experiment had different capacities for targeting genomic sequence (5 Mb, 1.6 Mb, and 3.3 Mb, respectively) downstream adjustments during analysis (described below) were made to ensure an equivalent amount of sequencing throughput was dedicated to each capture technology on a *sequence read per targeted bp* basis; that is, each capture platform is expected to have the same read depth, all else being equal.

#### Evaluation of design efficiency

To examine the efficiency with which each platform could design oligos or PCR amplicons to target regions of interest, an identical 5 Mb of genomic sequence (representing the largest commercial capture option at the time) was provided to each vendor for informatics-based targeting using on the vendor's standard informatics design strategy. We note that, at the time of study, only the Nimblegen Seqcap arrays had the capacity to target the entire 5 Mb region. As detailed below, only a subset of this 5 Mb, approximately 0.8 Mb, was able to be physically targeted by all three platforms (Table S7). The informatics design efficiency for each platform was then calculated as the fraction of bases out of the 5 Mb provided that could be targeted by oligo/amplicon design strategy employed by each vendor. We note that this *design efficiency* is independent of the actual *target enrichment efficiency*, which was empirically determined from sequencing data, as described below.

### Targeted Enrichment

Methods for each of the three enrichment platforms (Agilent, Nimblegen, Raindance) are provided in the subsections below. A total of 6 samples were captured and sequenced on a SOLiD slide “spot” (one sample per spot) by all three enrichment methods. These 6 individuals, referred to below as our “matched sample set”, are the focus of our primary analysis. The remaining set of 18 unique individuals were sequenced using the 3 enrichment techniques (6 individuals per method). This latter set of 18 samples is referred to as the “unmatched sample set,” and they are analyzed and reported separately throughout this manuscript.

#### Agilent SureSelect

Solution-based targeted enrichment by hybridization was performed at the UM/Center for Genome Technology according to the manufacturer's (Agilent) standard protocol for SOLiD library preparation. 3 ug of genomic DNA was sheared via sonication using the Covaris (S-Series) instrument. Biotynilated RNA oligonucleotide baits were hybridized with sheared DNA. Captured fragments were removed from solution via streptavidin-coated magnetic beads and subsequently eluted. The enriched fragment library was then subjected to PCR amplification using primers targeting the SOLiD anchors. Resulting libraries were quantified via Agilent Bioanalyzer before proceeding to SOLiD platform library preparation (described below).

#### Nimblegen SeqCap

Nimblegen SeqCap array capture (385 k feature array) was performed at the Nimblegen service center according to the company's standard SeqCap protocol. Briefly, genomic DNA was nebulized for 1 minute using 45 psi of pressure. Sheared DNA fragments were subsequently purified with the DNA Clean & Concentrator-25 Kit (Zymo Research) and Bioanalyzer (Agilent) traces were used to confirm a resulting fragment size distribution of 300 to 500 bp. At the time of this study, the Nimblegen captured protocol was optimized to target the Roche 454 sequencers. As a consequence, Roche 454 anchors were used in the capture procedure, resulting in additional protocol modifications (discussed in *Library Preparation and Sequencing* Section below). Following end-polishing of the genomic fragments, Nimblegen adaptors were ligated to the sheared genomic fragments. Ligated fragments were next hybridized to the 385 k SeqCap arrays within Maui hybridization stations, followed by washing and elution of array-bound fragments from the arrays within elution chambers (Nimblegen). Captured fragments were then subjected to 27 rounds of PCR amplification using primers targeting the Nimblegen linkers. Following elution, the capture efficiency was evaluated via q-PCR reactions. For additional details, see manufacturer's protocol (http://www.nimblegen.com/products/lit/SeqCap_UserGuide_Tit_Del_v1p0.pdf) and the resulting fragment library was shipped to the University of Miami Center for Genome Technology for further processing prior to SOLiD3.0 sequencing (described below.)

#### Raindance PCR Enrichment

Genomic enrichment via massively parallel PCR was conducted at Raindance Technologies, as previously described [6]. Resulting libraries were shipped to the UM/Center for Genome Technology for SOLiD library preparation (described below).

### SOLiD Library Preparation and Sequencing

For the purpose of this experiment, each captured sample was prepared for running on a single SOLiD3.0 slide octet “spot,” which was anticipated to yield between 25 and 40 million alignable 50 bp sequencing reads at the time of the experiment. Following enrichment, the Agilent SureSelect capture libraries proceeded directly to quantitation and emulsion PCR (described below). For both Raindance and Nimblegen target-captured libraries, fragment size requirements for the SOLiD required that the captured fragments first be concatenated via ligation so that they could be subjected to additional sonication in order to achieve a fragment length distribution of 150–200 bp for SOLiD sequencing. Following concatenation of the PCR products, 5 µg was quantitated using the Thermoscientific NanoDrop8000, aliquotted, and brought to volume in 100 µl of Ambion nuclease-free water for shearing with the Covaris E10. The sheared DNA was end-repaired and quantitated before attachment of the the SOLiD P1 and P2 adapters by ligation. The ligated template was loaded into a 2% agarose size-select Invitrogen E-gel and selected at the 150–200 base pair range. The size-selected libraries underwent nick translation and 3 cycles of library amplification. The Agilent 2100 Bioanalyzer DNA 1000 chip was used to confirm the libraries' fragment length and obtain a preliminary concentration of the stock aliquot. Quantitative PCR on the Roche Lightcycler480 was conducted using SOLiD adapter specific primers and Universal Probe Library (#149) (Table 2).

**Table 2:** Oligonucleotide sequences used, in conjunction with the Universal Probe Library # 149 (Roche), for qPCR of the SOLiD sequencing library.

Sequence	Sequence Name
5′ - CTGCCCCGGGTTCCTCAT TCTCT – 3′	*SOLiDLIBR*
5′ - GGCGGCGACCTCTCTATGGGCAGTCGGTGAT – 3′	*SOLiDLIBUPLF*

Using the concentration values obtained from the quantitative PCR, a 500 pM aliquot was prepared from the stock library and titrated to 0.9–1.0 pM for input into ABI 1.0 pM-scale emulsion reactions. Emulsion PCR was conducted using Applied Biosystems GeneAmp PCR system 9700 for 40 cycles of amplification. Following emulsion breaking and subsequent washing, enrichment for template beads was conducted using the SOLiD capture beads with P2 affinity. Beads lacking a template or a P2 adaptor were filtered out via centrifugation with glycerol. The P2-enriched beads were isolated from the upper glycerol layer, modified with a 3′ amino group for surface attachment, and prepared for deposit on the SOLiD slide. A single SOLiD octet “spot” was dedicated to each captured genomic sample.

### Data Analysis

#### Informatics pipeline

Following base calling, alignment and SNP calling was conducted using the ABI Bioscope vs. 1.2.1 (Applied Biosystems), with standard parameter settings for targeted resequencing. Sequencing reads from both platforms were randomly removed from the primary .*csfasta* and .*qual* files prior to further subsequent analyses in order to equalize the amount of sequencing throughput dedicated per basepair targeted. Coverage depth statistics were tabulated using in-house PERL scripts and based on read depth values obtained from diBayes output files (*ConsensusCalls.txt). Target enrichment efficiency for each platform was calculated as the number of base pair reads falling on an intended target coordinate vs. the total number of bases mapping anywhere within the genome. Summary statistics for coverage and associated plots were conducted using the R statistical programming environment. The distribution of coverage depth was visualized using kernel density plots, which provide a non-parametric means of examining the distribution of a random variable. [11], [12] We note that although probes for the X chromosome were targeted for enrichment, they were excluded from the analyses described below to simplify coverage comparisons across samples of different sex.

#### Handling of clonal reads

It is common practice to remove redundant sequence reads from clonal amplicons generated during library preparation by excluding those sequencing reads possessing identical start and stop positions. This procedure was not a viable option in our experiment, primarily because the concatenation and re-fragmentation of both Raindance and Nimblegen libraries resulted in the effective scrambling of start and stop position information. Hence, we could not fairly compare the three enrichment systems in this regard. Furthermore, as is the case with most custom targeted resequencing projects, the restricted amount of genome space being covered results in the replication of numerous start and stop positions by chance. Discarding these reads would result in the loss of significant amount of valid data. The impact of redundant reads would most likely have influenced allelic balance and genotype calling results. The observation that the three platforms exhibit little deviation in terms of these measures (see Results and Discussion) indicates that the enrichment platforms did not vary significantly with respect to fragment redundancy and associated complexity.

#### Read depth correlation across samples

Read depths associated with each targeted base position for all three platforms were extracted from the diBayes output (*.ConsensusCalls.txt). The resulting coverage data were filtered so that only individual nucleotide positions *targeted by all three capture platforms* were used for correlation analysis. Sample to sample correlation matrices for each platform separately were calculated using R statistical programming environment. We note that correlation statistics were only conducted for the six samples for which sequence was obtained using all three targeted enrichment techniques.

#### Genotype concordance

Genotype calls derived from the Bioscope 1.2.1 diBayes module (Applied Biosystems) were compared with data from Illumina 1M Infinium GWAS chip for five individuals for which prior genotype information was available. Concordance was defined and calculated as the total number of matching genotypes vs. all valid comparisons. Valid comparisons were defined as those where *a)* Illumina genotype data was present for the individual at the base position and *b)* the sequencing data for the corresponding position had a minimum coverage depth of 20×. We set a minimum coverage depth requirement to reduce the impact of sampling variance on genotype calling and focus primarily on how platform specific differences in allele ratio balance and/or quality. For all comparisons involving raindance enrichment, base positions corresponding to primer locations were excluded from the analysis.

#### Allelic balance at heterozygous loci

For the purpose of this study, we define allelic bias as the deviation from the expected 50/50 allele ratio at a diploid heterozygote loci. To investigate allelic bias resulting from enrichment procedures, the observed frequency of the non-reference allele at heterozygous loci were recorded across all loci previously determined to be heterozygous within an individual based on Illumina 1M genotyping data.

## Results and Discussion

### Target Design Efficiency

We first sought to determine the relative efficiency with which the three capture platforms could *design* capture assays across our region of interest using their standard probe/primer design methodology. Due to the different oligonucleotide lengths employed by Agilent and Nimblegen, flexibility in PCR primer placement by Raindance, and differences in the propriety informatics design strategy employed by each vendor, it was expected that some genomic regions would be more or less amenable to each vendor's design process due to variation in local repetitive DNA content, local GC content, and/or local secondary structure. For the comparison of target design efficiency, an identical set of five 5 Mb, comprised of exons and other conserved regions within gene transcripts (described in methods) were provided as input to each vendor's standard informatics platform for target design. Design efficiency was estimated as the total bp covered by designed probes (or amplicons) divided by the total bp of “regions of interest” provided for targeting. The Agilent design process, as implemented in eArray using default parameters, was achieved probe designs covering 89% of the requested 5 Mb of genomic surface area. The Nimblegen design pipeline achieved targeting of 91%, and the Raindance design process achieved 97% design efficiency. Probe and amplicon designs for each enrichment platform are provided in Tables S3,S4, and S5. The similar performance of the Agilent and Nimblegen design procedures was anticipated, as both platforms use an oligonucleotide hybridization-based approach and are thereby subject to similar constraints for oligonucleotide placement. The elevated design efficiency of Raindance is attributable to their ability to adjust primer position and amplicon length to accommodate repetitive sequence and other potentially problematic features, such as local extremes of GC content.

### Efficiency of On-Target Enrichment

We next examined the fraction of on-target bases sequenced following each targeted enrichment technique. The percentage of on-target bp that are sequenced has considerable influence on how much sequencing must be dedicated to each sample within a given study design, directly impacting project costs and timelines. Although sequence in the immediate vicinity of targeted regions can often be of interest, off-target sequencing is largely a waste of valuable sequencing throughput. We defined on-target enrichment efficiency as the fraction of total number of *mapped nucleotides* that overlapped a targeted nucleotide, divided by the total number of nucleotides mapping anywhere in the genome. For the purpose of enrichment efficiency, we compared the 6 matched samples, where were independently enriched, as described in methods, and sequenced on SOLiD 3.0 platform octet slides (18 octets “spots” in total). Of the initial 5 Mb used in the target design efficiency examination above, each capture platform targeted the fraction of the target list (in list order) that the commercial option was physically capable of targeting at the time of the experiment. In the case of Nimblegen SeqCap, this was the entire 5 mb of regions. Agilent SureSelect was capable of targeting the first 3.3 Mb of the 5 Mb total, and Raindance targeted 1.6 Mb of the total. After final design and library production, ∼1 Mb of genomic positions were physically targeted by all three platforms. All comparative platform analyses described below was conducted using only those base positions targeted by all three platforms. Since these shared positions represent a large and effectively random sampling of all positions targeted for each platform, metrics for bases outside the shared (i.e. platform overlap) positions are not appreciably different from shared regions and are therefore not shown. On target efficiency for the matched sample set (n = 6 samples repeated across each platform) and the unmatched data set (n = 6 different samples per enrichment platform) is provided in Tables 3 and 4. We note that our criteria for what counts as an on-target base is strict, in the sense that the sequence immediately flanking the targeted regions was excluded. This approach allowed a more fair comparison with the Raindance method, which does not benefit from the pull-down of sequence adjacent to probe regions. As further discussed below, Raindance performance on the unmatched sample set was markedly lower than observed for the matched sample set, primarily due to an outlier sample with low (38%) on-target efficiency.

**Table 3:** Percent on-target, matched sample sets (N = 6).

SampleID	Nimblegen	Agilent	Raindance
*Mean*	53.33	60.79	52.50
*Median*	53.35	61.45	49.90
*Range*	49.64–57.31	56.45–63.09	44.71–63.57
*Std Dev*	2.91	2.46	7.11

**Table 4:** Percent on-target, unmatched sample sets (N = 6).

SampleID	Nimblegen	Agilent	Raindance
*Mean*	55.56	61.64	46.18
*Median*	56.13	62.56	45.28
*Range*	53.29–57.75	54.48–68.55	38.05–52.65
*Std Dev*	1.86	5.09	5.65

### Coverage Depth and Uniformity of Sequencing Coverage

**Figure 1:**
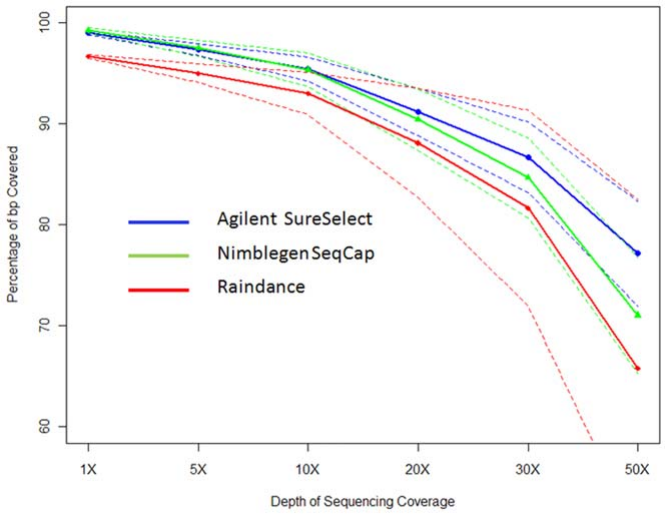
Depth of sequencing coverage of matched sample set (N = 6 unique samples). Percent of on-target bases (y-axis) covered at a given sequence depth (x-axis). On target percentage calculated as the fraction of nucleotide bases falling on targeted regions divided by the total number of nucleotides mapping anywhere in the genome. Thick lines represent average coverage for each platform (Agilent Surelect = blue circles; Nimblegen SeqCap = green triangles; Raindance parallel PCR = red diamonds). Dashed lines represent two standard deviations above and below the average for each platform.

**Figure 2:**
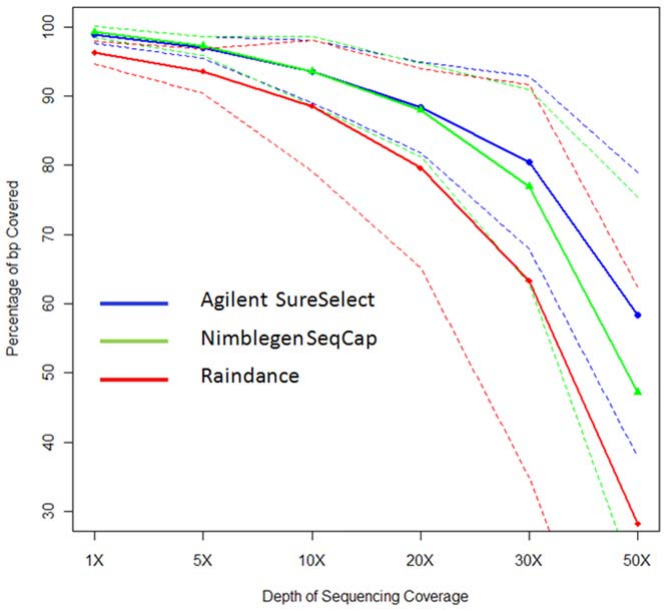
Depth of sequencing coverage of matched sample set (N = 6 samples (per method)). Percent of on-target bases (y-axis) covered at a given sequence depth (x-axis). On target percentage calculated as the fraction of nucleotide bases falling on targeted regions divided by the total number of nucleotides mapping anywhere in the genome. Thick lines represent average coverage for each platform (Agilent Surelect = blue circles; Nimblegen SeqCap = green triangles; Raindance parallel PCR = red diamonds). Dashed lines represent two standard deviations above and below the average for each platform.

**Figure 3:**
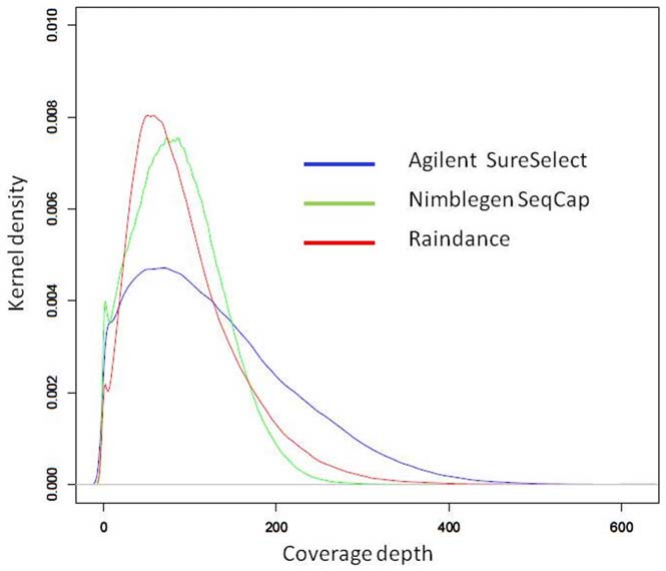
Kernel density of coverage depth. Depicts kernel density function for the three enrichment platforms studied. The set of coverage depth values at each target position were pooled across all individuals from the matched sample set and the frequency of values at each depth were used to calculate the density function.

The depth of sequence coverage at targeted positions is clearly a key consideration for targeted resquencing. Depth of sequencing directly influences one's ability to adequately infer genotypes. Given that the mean and median of coverage depth across positions generally fails to provide a useful metric due to extensive variation across genomic loci, one practical measure that researchers rely upon is the fraction of target positions that are covered at greater than or equal to a given depth (e.g. 20×). To address the fact that Agilent SureSelect and Raindance enrichment data contained a higher ratio of sequencing throughput per base pair targeted (i.e. due to the fact that they targeted less total genomic space but the enriched samples were sequenced on the same “octet” spot format as the Nimblegen platform), we imposed an artificial “handicap” on the Agilent and Raindance platform data by randomly removing reads to equalize the amount of sequencing throughput dedicated per basepair targeted. Figures 1 and 2 show the percentage of targeted basepairs covered at a given coverage depth for both the matched and unmatched samples sets respectively. Overall, Agilent exhibits superior coverage performance, with percent of sites covered at a given depth falling off more slowly than observed for either Raindance or Nimblegen. Agilent and Nimblegen performance were similar at 20× coverage depth, with differences primarily emerging at 30× coverage and above. We note that Nimblegen suffered coverage loss in our experiments due to both the addition of 454 anchors during the enrichment protocol, which resulted in less sequence throughput being dedicated to each genome, and due to post-enrichment concatenation and subsequent re-shearing of products. Hence, protocol adjustments that circumvented either of these steps would be expected to bring results closer in line with Agilent. The overall coverage depth performance is similar in both the matched and unmatched sample sets. Again, raindance performance is notably lower in the ummatched compared to the matched set; this is largely attributable to one outlier sample that exhibited a lower on-target efficiency. To achieve a better view of how coverage depth was distributed for each of the enrichment methods, we generated kernel density plots for coverage depths across all targeted basepairs across all samples (Figure 3). Data from all individuals for a given enrichment platform was pooled prior to plotting the density function. Interestingly, both Nimblegen and Raindance exhibit tighter coverage depth distributions, with less variation about the mean. The agilent distribution is broader, with its tail shifted towards the higher coverage depths.

### Consistency of Capture Results

**Figure 4:**
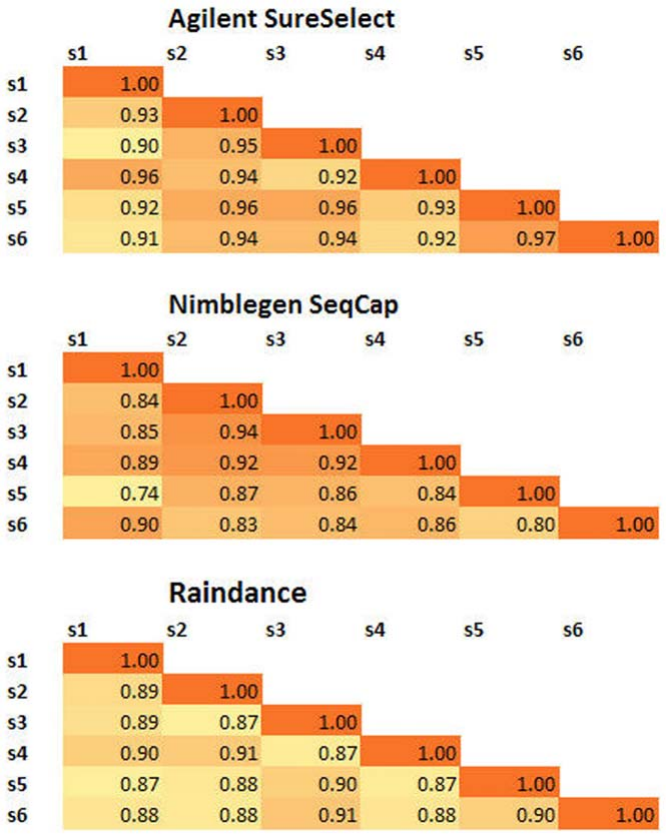
Pearson correlation matrix for coverage depth. Pearson correlation matrix depicting sample to sample comparisons for each independent platform. Only matched samples (i.e. individual samples that were separately enriched on across all three platforms) were used for this analysis. Cells with higher correlation values appear in darker shades.

When performing targeted resequencing on a population of samples, the consistency of results across independent DNA samples is a key consideration. A high level of sample to sample correlation of coverage depth across target positions facilitates the process of determining how much sequencing throughput is required to achieve a given level of coverage across a resequencing experiment. We examined the sample to sample correlation of coverage depths across individuals in the matched set for each of the targeted enrichment techniques. The pearson correlation matrix for the six matched samples is given in Figure 4. Coverage depth correlations for the same site across individuals was highest for Agilent, followed by Nimblegen and Raindance. Depth of coverage correlation across platforms (i.e. Agilent vs. Raindance) was substantially lower, although, as expected, the two hybridization-based procedures (SureSelect and SeqCap) exhibited higher similarity to each other than the amplicon-based method (Raindance).

### Allelic Balance at Heterozygote Loci

All else being equal, the expected frequency within the sequence fragment data for each allele at a diploid heterozygous loci should be 0.5. Several factors can result in deviations from this expectation. These include biases in the target enrichment process favoring one alle over another, biases in amplification during sequencing library preparation, biases in sequence alignment favoring reference alleles, as well as the presence of non-unique sequence (e.g. interspersed repeats or structural variation) that comprimise alignment. To assess the distribution of allele frequencies at heterozygous loci, we examined all base positions in each individual where the Illumina array data indicated a position was heterozygous. The distribution of observed frequency of the non-reference allele at each position was compared to the expected value of 0.5. While there was some tendency for the reference allele to be over-represented in comparison to the non-reference allele (discussed below), there were no appreciable differences among enrichment platforms in the average non-reference allele frequency (NAF) or the variance of NAF. Across the five samples examined for each platform, average NAF was 0.41 for Agilent, 0.39 for Nimblegen, and 0.39 for Raindance. Variance was 0.004, 0.005, and 0.007 respectively. Despite the similarities across enrichment methods, our results indicated a consistent negative bias (∼10%) in the observed frequency of the non-reference allele for all three platforms, which we suspected was a sequence alignment issue on account of its consistency across all three enrichment platforms. Briefly, when one or more additional errors were present on a fragment, the addition of a mismatch to the human reference due to the presence of a legitimate SNP occassionally results in a fragment falling below the mismatch threshold and failing to align at the location. Applied Biosystems (personal communication) confirmed that this reference bias exists in the current implementation of the Bioscope alignment algorithm, and efforts are underway to mitigate this isue in future implementations. As indicated by the genotype concordance below, however, this bias was not substantial enough to greatly impact genotype calling accuracy at the positions we examined.

### Genotype Concordance

In order to assess the potential impact of enrichment technologies on downstream genotype concordance, we compared SOLiD sequencing data from each platform with previously obtained Illumina 1M infinium array data. Concordance was simply defined as the fraction of matching genotypes out of the total valid comparisons. To minimize the impact of sampling variance on results, valid comparisons were those that had a minimum of 20× coverage. While increased sampling variance, due to low site coverage, could be reflective of poor capture performance and/or insufficient sequencing throughput, here, we wanted to focus on how biases in hybridization and/or amplification associated with each technique might have skewed allele representation and impacted final genotype calling. As indicated in Table 5, genotype concordance was comparable across all platforms, suggesting that the enrichment platforms did not introduce a substantial bias in allele representation that impacted genotype calling.

**Table 5:** Genotype concordance.

Sample ID	matches/comparisons	Agilent concord.	matches/comparisons	Nimblegen concord.	matches/comparisons	Raindance concord.
s2	186/187	0.99	191/192	0.99	170/173	0.98
s3	190/190	1.00	191/192	0.99	167/172	0.97
s4	188/188	1.00	192/192	1.00	177/179	0.99
s5	186/187	0.99	190/191	0.99	176/178	0.99
s6	186/186	1.00	188/188	1.00	177/180	0.98
	**mean**	0.998		0.997		0.983
**Total genotype comparisons**		**938**		**955**		**882**

These data represent a snapshot in time of what has proven to be a rapidly changing field of genomic target enrichment. Since the time of these experiments were carried out, protocol modifications have been made by Raindance and Nimblegen, and additional genomic enrichment options, including an in-solution hybridization option from Roche-Nimblegen, have become available on the market. Nevertheless, the data presented here provide useful information that will aid in gauging the performance of different capture approaches and assessing how generalizable enrichment method performance will be across multiple sequence platforms. While we find that each enrichment platform exhibited strengths in one or more dimensions, the overall performance of Agilent custom capture was superior across the majority of measures. In particular, we observed higher on-target efficiency with Agilent, which ultimately resulted in increased coverage depth performance. We also observed increased sample to sample consistency, as measured by correlation of read depth across samples. Raindance demonstrated a distinct advantage in the ability to target a larger percentage (97%) of our regions of interest due to its flexibility with primer placement, allowing more repetitive content to be targeted. This can be a key consideration, particularly for diagnostic resequencing or other scenarios where contiguous coverage of gene targets is imperative. As indicated in Figure 3, both Raindance and Nimblegen exhibited tighter sequence coverage depth distributions around the mean as compared to Agilent, but the benefits of these tighter distributions were outweighed by lower on-target efficiency that was observed for these platforms in our experiments. Sequencing results using all three target enrichment methods studied exhibited excellent concordance with known genotypes, suggesting no systematic biases were present that compromised accurate genotype calling.

## Supporting Information

Table S1 **Positions targeted for genomic enrichment by random selection from across the genome.**
(XLSX)

Table S2 **Positions targeted for genomic enrichment based on disease relevance.**
(XLSX)

Table S3 **Final target regions sent to vendor or earray website for probe design.**
(XLSX)

Table S4 **Probe locations from Agilent earray design.**
(XLSX)

Table S5 **Raindance amplicon design.**
(XLSX)

Table S6 **Probe locations from Nimblegen design.**
(XLSX)

Table S7 **Positions (hg18) of basepairs targeted by all three capture methods.**
(XLSX)
